# Overexpression of COL11A1 by Cancer-Associated Fibroblasts: Clinical Relevance of a Stromal Marker in Pancreatic Cancer 

**DOI:** 10.1371/journal.pone.0078327

**Published:** 2013-10-23

**Authors:** Carmen García-Pravia, José A. Galván, Natalia Gutiérrez-Corral, Lorena Solar-García, Eva García-Pérez, Marcos García-Ocaña, Jokin Del Amo-Iribarren, Primitiva Menéndez-Rodríguez, Juan García-García, Juan R. de los Toyos, Laureano Simón-Buela, Luis Barneo

**Affiliations:** 1 Pathological Anatomy Service, Hospital Universitario Central de Asturias (HUCA), Oviedo, Spain; 2 Instituto Universitario de Oncología del Principado de Asturias (IUOPA), Oviedo, Spain; 3 Department of Surgery, School of Medicine and Health Sciences, University of Oviedo, Oviedo, Spain; 4 General Surgery Service, Hospital Universitario Central de Asturias (HUCA), Oviedo, Spain; 5 Biotechnological and Biomedical Assays Unit, Technical-Scientific Services, Oviedo, Spain; 6 Progenika Biopharma, S.A., Science and Technology Park of Zamudio, Derio, Spain; 7 Immunology Area, School of Medicine and Health Sciences, University of Oviedo, Oviedo, Spain; 8 Oncomatrix, S.L., Science and Technology Park of Zamudio, Derio, Spain; Centro Nacional de Investigaciones Oncológicas (CNIO), Spain

## Abstract

**Background:**

The collagen11A1 (COL11A1) gene is overexpressed in pancreatic cancer. The expression of COL11A1 protein could be involved in desmoplastic events in pancreatic cancer, but an antibody that specifically stains the COL11A1 protein is not currently available.

**Methods and findings:**

A total of 54 pancreatic ductal adenocarcinomas (PDAC), 23 chronic pancreatitis (CP) samples, and cultured peritumoral stromal cells of PDAC (passages 3-6) were studied. Normal human pancreas tissue samples were obtained through a cadaveric organ donation program.

1) Validation of COL11A1 gene overexpression by q-RT-PCR. Findings: the expression of COL11A1 gene is significantly increased in PDAC samples vs. normal and CP samples.

2) Analysis of COL11A1 by immunohistochemistry using highly specific anti-proCOL11A1 antibodies. Findings: anti-proCOL11A1 stains stromal cells/cancer-associated fibroblasts (CAFs) of PDAC but it does not stain chronic benign condition (chronic pancreatitis) stromal cells, epithelial cells, or normal fibroblasts.

3) Evaluation of the discrimination ability of the antibody. Findings: anti-proCOL11A1 immunostaining accurately discriminates between PDAC and CP (AUC 0.936, 95% CI 0.851, 0.981).

4) Phenotypic characterization of proCOL11A1+ stromal cells co-staining with mesenchymal, epithelial and stellate cell markers on pancreatic tissue samples and cultured peritumoral pancreatic cancer stromal cells. Findings: ProCOL11A1+ cells present co-staining with mesenchymal, stellate and epithelial markers (EMT phenotype) in different proportions.

**Conclusions/Significance:**

Detection of proCOL11A1 through immunostaining with this newly-developed antibody allows for a highly accurate distinction between PDAC and CP. Unlike other available antibodies commonly used to detect CAFs, anti-proCOL11A1 is negative in stromal cells of the normal pancreas and almost absent in benign inflammation. These results strongly suggest that proCOL11A1 is a specific marker for CAFs, and thus, anti-proCOL11A1 is a powerful new tool for cancer research and clinical diagnostics.

## Introduction

Pancreatic ductal adenocarcinoma (PDAC) represents the fourth leading cause of death from cancer in men and women. The 5-year survival rate is less than 5% and average survival time is 6 months after the initial diagnosis. Even in patients who undergo resection, long-term survival rates remain extremely poor [1]. At the present time, there are no early diagnosis methods or effective therapies for use against this type of tumor. Despite progress having been made in its diagnosis and treatment, pancreatic cancer continues to have the worst prognosis of all solid malignant tumors. Pancreatic cancer is the paradigm of advanced neoplastic disease: independently of the TNM stage, the majority of patients present with disseminated disease in the early phases [2]. Furthermore, pancreatic cancer is resistant to chemo- and radiotherapy [3].

Pancreatic carcinoma is characterized by a desmoplastic reaction involving cellular and acellular components, such as fibroblasts (activated or resting), myofibroblasts, pericytes, pancreatic stellate cells, immune cells, blood vessels, the extracellular matrix, and soluble proteins such as cytokines and growth factors [4,5]. This heterogeneous stroma influences multiple aspects of PDAC and seems to promote tumor growth, invasion, and resistance to chemotherapy [6-9]. 

Chronic pancreatitis is an inflammatory disease characterized by irreversible and progressive destruction of the organ, resulting in exocrine and endocrine insufficiency. The lost parenchyma is replaced by dense fibrous tissue with infiltrating leukocytes and ductular hyperplasia. Chronic pancreatitis significantly increases the risk of developing pancreatic cancer [10-12], which suggests that chronic inflammation within the pancreas may be a predisposing factor to the development of cancer. The causative link between chronic inflammation and cancer was described two centuries ago by Marjolin [13], but the inflammatory mediators that lead to the development of cancer remain undefined.

Among the tumor-associated matrix collagens, fibrillar collagens are the most conspicuous. Collagens are synthesized as procollagens by fibroblasts. These procollagens have a main central triple-helical domain, designated as α1, α2, and α3, and coded by specific gene sequences. Once secreted to the extracellular milieu, these procollagens are cleaved, and then the mature collagen molecules assemble extracellularly in fibrils. In normal tissues, collagen types I, II and III are the main major fibrillar collagens, while collagens V and XI are less abundant minor fibrillar collagens [14]. Collagens V and XI share a 75% homology at their amino acid sequence level. Procollagens α1 of types V and XI are coded by COL5A1 and COL11A1 genes, respectively.

Studies of the fibroblasts in the vicinity of the tumor, the so-called cancer-associated fibroblasts (CAF), have demonstrated their role in stimulating tumor progression [15-20]. The characteristics of the CAFs have been investigated in depth, showing that their genotypic expression, growth pattern, migratory behavior and secretion of growth factors differ from those of normal fibroblasts [15,21]. However, researchers do not have specific tools to differentiate CAFs from inflammatory fibroblasts. Vimentin (VIM) and alpha-smooth muscle actin (αSMA) are often used to identify CAFs, but these bio-markers are not specific, since they stain inflammatory fibroblasts and other cells as well. We have previously identified 116 genes that were overexpressed in PDAC using DNA microarrays [22] (see File S2). We found genes of the extracellular matrix whose expression was increased compared to normal and chronic pancreatitis (CP) tissues. One of the most significantly and consistently overexpressed genes was COL11A1 (Table S1 in File S1). Given the lack of a reliable commercial antibody, we generated a rabbit polyclonal antiserum to a highly specific amino acid stretch of human proCOL11A1 in order to assess the protein level expressed in pancreatic cancer [23]. Subsequently, we developed a monoclonal antibody [24], highly specific for human proCOL11A1, which clearly marks these peritumoral stromal cells.

In this study we attempted to confirm the overexpression of COL11A1 gene in PDAC. In addition, we evaluated the clinical utility of the immunodetection of proCOL11A1 in PDAC and CP tissues. Finally, we explored the phenotypic characteristics of COL11A1-expressing cells within the pancreatic stroma.

## Materials and Methods

### Patient characteristics and tissue sampling

The immunohistochemistry studies with anti-proCOL11A1 pAb were performed on 54 PDAC and 23 CP (20 alcoholic, 2 autoimmune, 1 biliary) historical paraffin-embedded samples obtained from surgical specimens from patients who underwent surgery at the Hospital Universitario Central de Asturias, Oviedo, Spain. The mean age of the patients with PDAC was 65 ± 9 years (46-81 years old) and, 56 ± 12 years (25-73 years old) of the patients with CP. The gender ratio (M/F) was 37/17 for the PDAC patients, while male predominance was even higher in the CP patients (22/1). The distribution of PDAC tumor stage according to the TNM classification (AJCC Cancer Staging Manual. 7th ed. 2010) was: IA, 13%; IB, 17%; IIA, 19%; IIB, 37%; III, 6%; IV, 9%. The neoplastic grading was: G1, 20%; G2, 66%; G3, 12%; G4, 2%. The anti-proCOL11A1 mAb was applied to 69 (51 PDAC and 18 CP) of the previous cases. Freshly removed PDAC, CP and normal pancreas tissue samples were immediately snap-frozen in liquid nitrogen in the operating room and stored at -80°C until processing. The normal human pancreas tissue samples were obtained through a cadaveric organ donation program (6 cases, all 41-76 year old males) and were used for q-RT-PCR. Fresh samples were used to isolate and culture the fibroblasts. Q-RT-PCR studies were applied to frozen PDAC and CP samples. This study complies with the Helsinki Declaration and was approved by the Hospital Universitario Central de Asturias ethical committee (Project n° 42/12). All patients signed consent forms indicating their willingness to participate and their understanding of the procedure and general aim of the study.

### Quantitative RT-PCR of COL11A1

Total RNA was isolated from pancreas biopsies using TRIzol reagent (Life Technologies), and cDNA was synthesized with the reverse transcriptase enzyme SuperScript II RNAse (Life Technologies). All PCR reactions were run in duplicate on a LightCycler 2.0 (Roche) using the FastStart DNA master SYBR Green I kit (Roche). Primer sequences were as follows: COL11A1 (target gene): forward 5’ TGGTGAT CAGAATCAGAAGTTCG 3’, reverse 5’ AGGAGAGTTGAGAATTGGGAATC 3’; Ribosomal protein L10 (reference gene): forward 5’ TGCGATGGCTGCACACA 3’, reverse 5’ TCCCTTAGAGCAACCCATACAAC 3’. The efficiency of the PCR reactions was calculated using reference curves with serial dilutions of cDNA. The ratios of normalized gene expression values were determined using the relative quantification equation which corrects for differences in PCR efficiency [25]. Finally, gene expression data were compared between tumor and control samples using the Mann-Whitney U test.

### Rabbit polyclonal antiserum to the variable region of human procollagen 11A1 (anti-proCOL11A1 pAb)

We have previously described the generation of this antiserum [23]. Briefly, after the recombinant COL11A1-TGST fusion protein was constructed, expressed, and purified, a New Zealand white rabbit was injected intramuscularly at 2-week intervals with 2 ml of immunogen emulsion of the purified COL11A1-T-GST fusion protein (see File S2). The antiserum obtained by cardiac puncture was depleted of the anti_GST reactivity. The IgG fraction was purified using Protein ASepharose.

### Mouse monoclonal antibody to human procollagen 11A1 (anti-proCOL11A1 mAb)

The methodology for generating the anti-human proCOL11A1 monoclonal antibody and for the characterization thereof has been published [24]. In summary, the purified COL11A1-T-GST fusion protein was used to hyperimmunize BALB/c mice. Using Sp2/0 myeloma cells as fusion partner, B-cell hybridomas were generated by standard methods. The antibody, DMTX1, was supplied by Oncomatrix, S.L., Derio, Spain (Ref# P0002, lot#200212).

### Establishment of cell cultures

Samples were obtained from the tumor area, peritumoral area and normal area using different surgical blades to avoid contamination. Representative aliquots were histologically examined. The samples were transported in RPMI-1640 Medium (Gibco, Invitrogen) supplemented with amikacin and vancomycin (Normon Laboratories, Madrid, Spain, both at 40 μg/ml) to the culture laboratory where they were cut into smaller fragments using surgical scissors. The fragments obtained were enzymatically digested with collagenase (Type I 2 mg/ml, Sigma) for 1-2 hours. After the digestion, the collagenase solution was centrifuged at 400 g for 10 minutes. The pellet was resuspended in a fibroblast culture medium (Dulbecco’s modified Eagle’s medium (Gibco, Invitrogen) supplemented with 10% fetal calf serum (FCS, Gibco, Invitrogen), amikacin and vancomycin (both at 40 μg/ml). The tissue fragments that had not been digested with collagenase underwent a second digestion with 0.05% trypsin and 0.02% EDTA (T/E, Gibco, Invitrogen, Barcelona, Spain) for 30-60 minutes. The removed T/E was inactivated with serum-containing culture medium (DMEM + 10% FCS) and centrifuged at 400 g for 10 minutes. The pellet was resuspended in fibroblast culture medium. Cells obtained from collagenase digestion and trypsin digestion were seeded in six-well plates using fibroblast culture medium and maintained at 37°C in a 5% CO2 incubator. The medium was changed every 3 days. When the primary culture was confluent, the cells were washed twice with PBS and treated with T/E until they were detached. Then, the T/E was neutralized with culture medium and centrifuged at 400 g for 10 minutes to recover the cells. All stromal cells were used at early passages (passages 3-6). The cell purity of stromal cells was assessed by morphology and by immunostaining for vimentin.

### Immunohistochemistry and double immunostaining in pancreatic tissue resection

The tissue obtained from the pancreas samples was fixed in a 10% formaldehyde solution, paraffin embedded, cut 3 μm thick and stained with H&E for histological examination. Antigen retrieval was performed by heating the samples in *PTLink* (DakoCytomation, Denmark) in buffer solution at high pH for 20 minutes. Endogenous peroxidase activity was blocked with *Peroxidase Blocking Reagent* (DakoCytomation, Denmark) for 5 minutes. The samples were incubated at 37°C with the primary antibodies described in Table 1, incubated with the EnVision HRP Flexsystem (DakoCytomation, Denmark) for 30 minutes at room temperature, and stained with DAB (3-3´-Diaminobenzidine) (DakoCytomation, Denmark) for 10 minutes. Finally, they were counterstained for 10 minutes with hematoxylin (DakoCytomation, dehydrated and mounted in Entellan® (Merck, Germany). AntiproCOl11A1 pAb was assayed at 1:2000 in buffer S2022 (Dako).

**Table 1:** Antibodies used in immunohistochemical analysis.

**Primary Antibodies (species)**	**Clone**	**Commercial reference**	**Dilution**	**Incubation time (min)**
ProCOL11A1 (mAb)	1E8.33	(DMTX1) Oncomatrix	1:400	30
ProCOL11A1 (pAb)		Oncomatrix	1.2000	30
Alpha-smooth muscle actin (mAb)	1A4	Dako, Denmark	R-t-U	20
Desmin (mAb)	D33	Dako, Denmark	R-t-U	20
GFAP (mAb)	GA-5	Biogenex,The Netherlands	1:200	20
Citokeratin 7 (mAb)	OV-TL 12/30	Dako, Denmark	R-t-U	20
Vimentin (pAb)	C-20	Santa Cruz Biotech, Germany	1:600	10

(pAb) Polyclonal Rabbit ,(mAb) Monoclonal Mouse, R-t-U Ready to Use

To assess the coexpression of Pro-Col11A1 with mesenchymal (αSMA and VIM), epithelial (CK7), and pancreatic stellate cell (desmin, Glial Fibrillary Acidic Protein-GFAP-) markers, double immunostaining was performed using the ultra view Universal Alkaline Phosphatase Red detection kit (Ventana Medical Systems, Tucson, AZ) as red chromogen and DAB as brown chromogen. Previously, the samples had been incubated at 37°C with the primary antibodies described in Table 1.

### Immunocytofluorescence of cultured stromal cells and Confocal Microscopy

Cells were fixed in acetone (-20°C) for 10 minutes in the chamber slide. The cells were dried at room temperature and then were introduced into the wash buffer (Dako) for 30 minutes. The samples were incubated with the anti-proCOl11A1 mAb, (DMTX1, Oncomatrix) with Cytokeratin 7 (CK7) antibody and VIM antibody, to room temperature, under the conditions specified in Table 1. The secondary antibodies used were green anti-rabbit Alexa-488 (1:500, Invitrogen) and red anti-rabbit Alexa-546 (1:500, Invitrogen), for 1 hour at room temperature. Finally, the sections were mounted with mounting medium containing DAPI (Vector Labs). 

The colocalization (proCOL11A1 vs. VIM, proCOL11A1 vs. Ck7, proCOL11A1 vs. αSMA and VIM vs. CK7) was visualized and photographed using a Leica TCS SP2 confocal microscope with 63X and 100X oil immersion objectives, using the following sources of illumination for each fluorochrome excitation: Argon/Krypton laser (488 nm), Helium/Neon laser (546 nm) and blue-violet Diode (405 nm).

### PDAC vs. CP immunohistochemistry assessment

Assessment was carried out in four regions selected as the best representatives of the desmoplastic reaction. Cases that presented 4 positive fields through the 10X objective were assigned the maximum score of 4, while negative fields were assigned the minimum score of 0. For the staining assessment, a 20X field in the largest area with the most intense staining (“hot spot”) was chosen. Cases with less than 1% positive cells in relation to the stromal surface were assigned a score of 0, cases with between 1 and 10% were assigned a score of 1, cases with between 10% and 50% were assigned a score of 2, and cases with more than 50% positive cells were assigned a score of 3. Total variation in staining was defined by multiplying the number of positive fields (0-4) by the field’s staining intensity (0-3), yielding a total score of 0-12. The immunostaining was double-blind scored by two pathologists (CGP and JGG). A series of 24 PDAC and 16 CP immunostained slides were also quantified by means of the QWin image analysis program (Leica) in order to compare them to the pathologists’ scores. Images of the largest area with the most intense staining were taken through the 20X objective of an Olympus BX61 microscope and recorded on an Olympus Dp70 camera.

### Statistical data analysis

Assuming unequal variances, we applied the Welch test to determine the significance of the differences in the image data between PDAC and chronic pancreatitis samples. The ANOVA test was also applied. The correlation between various imaging parameters was assessed using the Spearman Rank correlation test. The sensitivity and specificity of proCOL11A1 was depicted as a receiver operating characteristic (ROC) analysis. The accuracy (i.e. correctly classified cases) was calculated. The position of the cut-off in the curve will determine the number of true positives, true negatives, false positives and false negatives. The criterion value is the cut-off corresponding to the highest accuracy (minimal false negative and false positive results). The sample size used in our immunohistochemical study (n=77) allowed us to achieve statistical significance (alpha=0.05) for an AUC=0.9 under the null hypothesis of an AUC=0.5, with a statistical power of 90%. All the analyses were implemented using the Statistical Package for the Social Sciences (SPSS 13) or MedCalc v9.4.1.0.

## Results

### Q-RT-PCR of COL11A1 and immunohistochemistry of pancreatic tissues with anti-proCOL11A1

**Figure 1:**
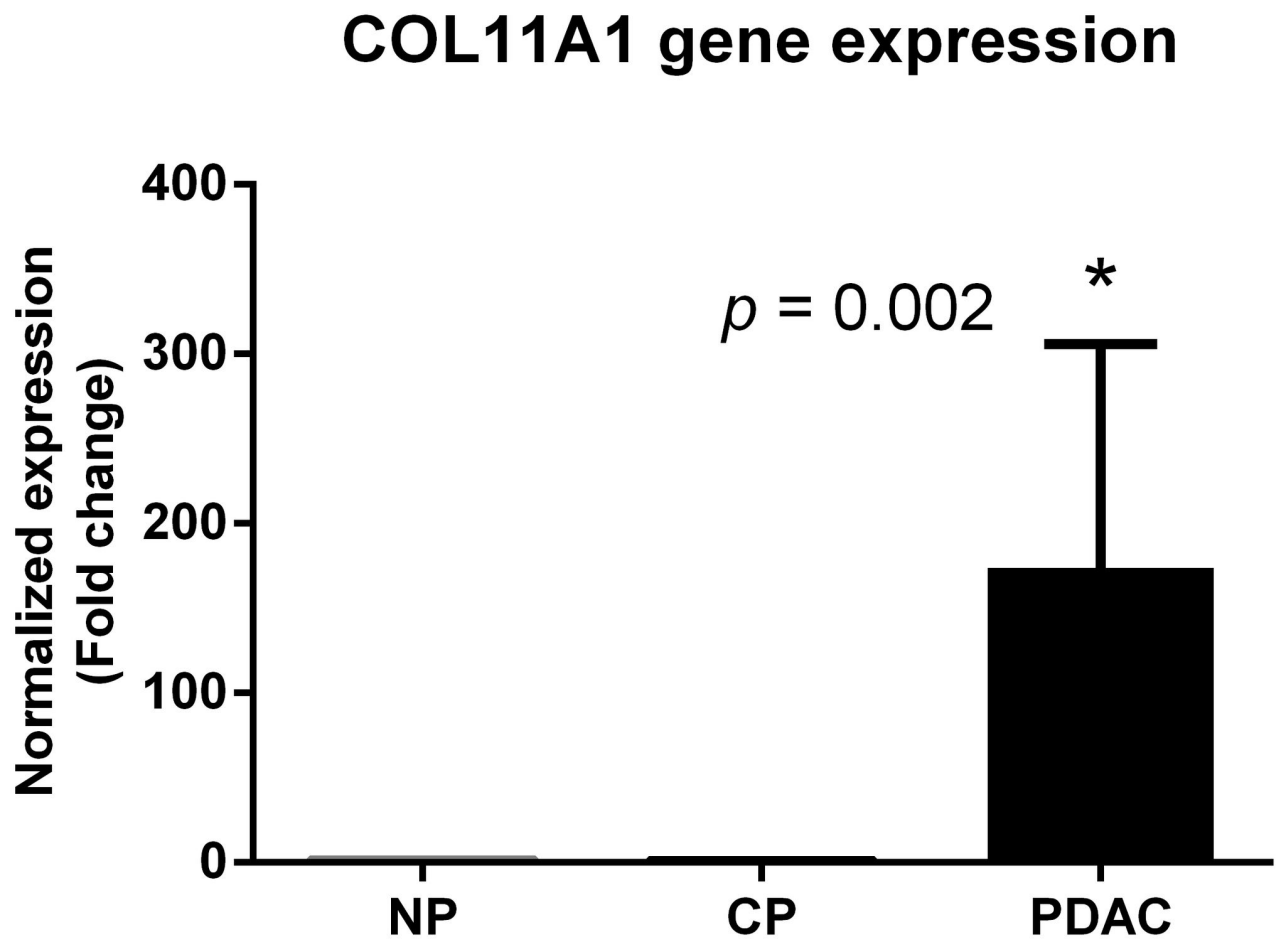
Normalized gene expression levels (log_2_-transformed fold change) in pancreatic ductal adenocarcinoma (PDAC) samples relative to normal pancreas (NP) and chronic pancreatitis (CP).

Pancreatic cancer cells express high levels of COL11A1 mRNA, as shown by quantitative RT-PCR (Figure 1). The results confirmed a significantly increased expression of the COL11A1 gene in PDAC samples vs. normal and CP samples (fold change: 174; SLR: 7).

**Figure 2:**
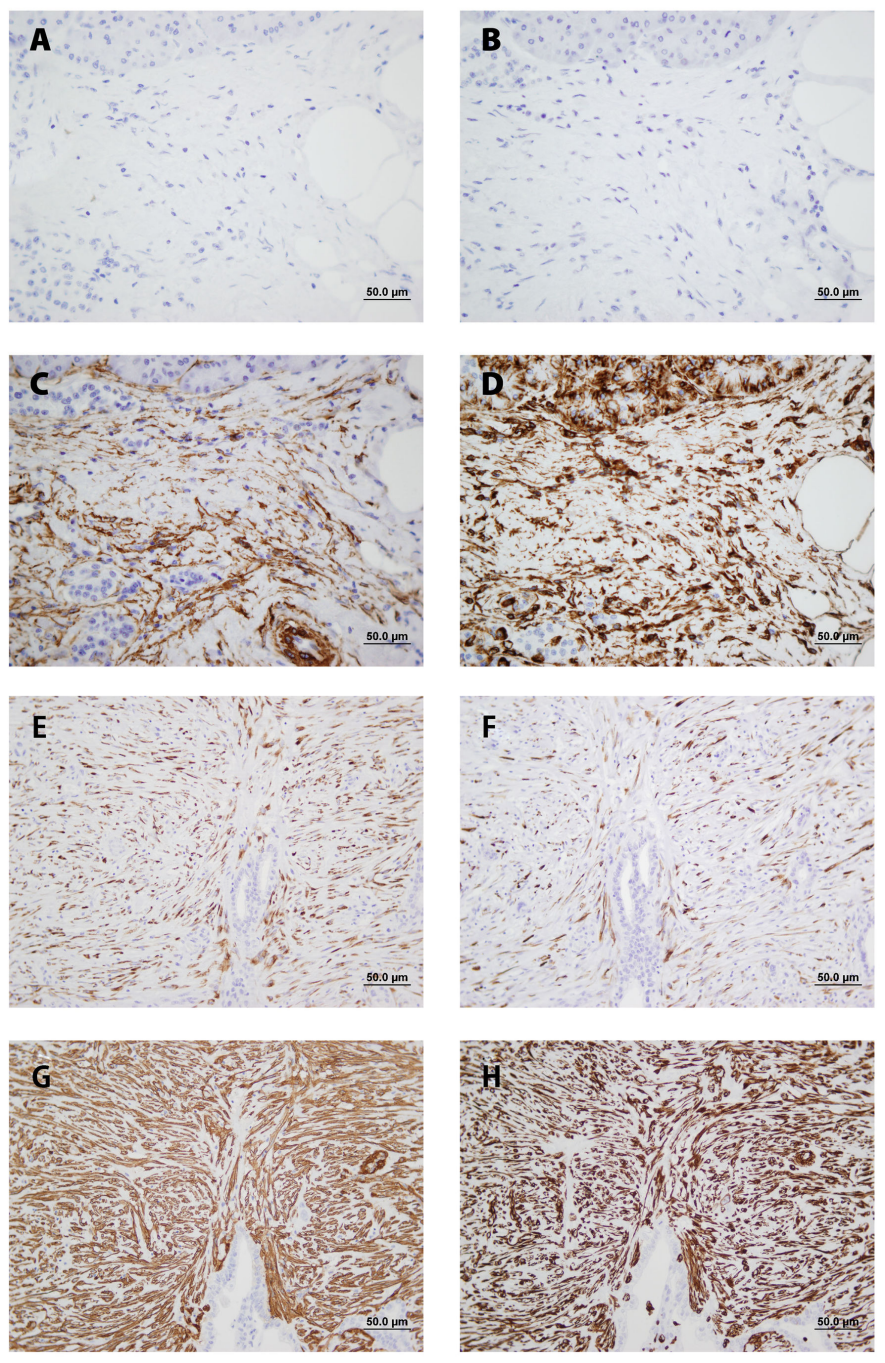
Comparative immunohistochemical profile of stromal cells of chronic pancreatitis (CP) and pancreatic ductal adenocarcinoma (PDAC) in serial sections. Stromal cells in CP were negative for anti-proCOL11A1 mAb (A) and desmin (B) whereas a substantial number of stromal cells of PDAC expressed anti-proCOL11A1 mAb (E) and desmin (F). In CP and PDAC the stain of stromal cells for αSMA (C and G, respectively) and VIM (D and H, respectively) were diffuse and non-selective. Anti-proCOL11A1 mAb (A and E), desmin (B and F), αSMA (C and G) and VIM (D and H) (all photomicrographs at ×400, Scale bar 50 μm).

All PDAC samples tested with anti-proCOL11A1 mAb and with antiproCOL11A1 pAb showed strong intracytoplasmatic labeling of tumor-surrounding desmoplastic stromal CAFs (Figure 2E; Figure S1D in File S1). The staining was not widespread but rather localized in restricted peritumoral areas, even where tumor cells were not seen. The morphological features of these fibroblast-like stromal cells were compatible with those of myofibroblasts (Figure SlE, F in File S1). Extracellular staining was never observed. In contrast, the expression of proCOL11A1 in chronic pancreatitis samples was either absent (Figure 2A; Figure S1B in File S1) or very low and restricted to a few stromal cells. CP stroma showed fibrotic and inflammatory changes with no remarkable myofibroblastic population. Normal pancreas and epithelial tumor cells, on the other hand, did not show any staining at all with anti-proCol11A1 (Figure S2 in File S1). Normal pancreas showed staining with VIM and αSMA, but did not stain with GFAP. The majority of CP (Figures 2C, D) and PDAC (Figures 2G, H) stromal cells showed strong intracellular staining with VIM and αSMA. The staining with desmin was intense in PDAC (Figure 2F) samples but nonexistent in CP (Figure 2B) samples. Figure S3 in File S1 shows positive staining with anti-proCOL11A1 mAb (score 4) and desmin in a case of autoimmune pancreatitis; in addition, intense positivity with VIM and αSMA, and negativity with GFAP is observed.

### Characterization of pancreatic CAFs

**Figure 3:**
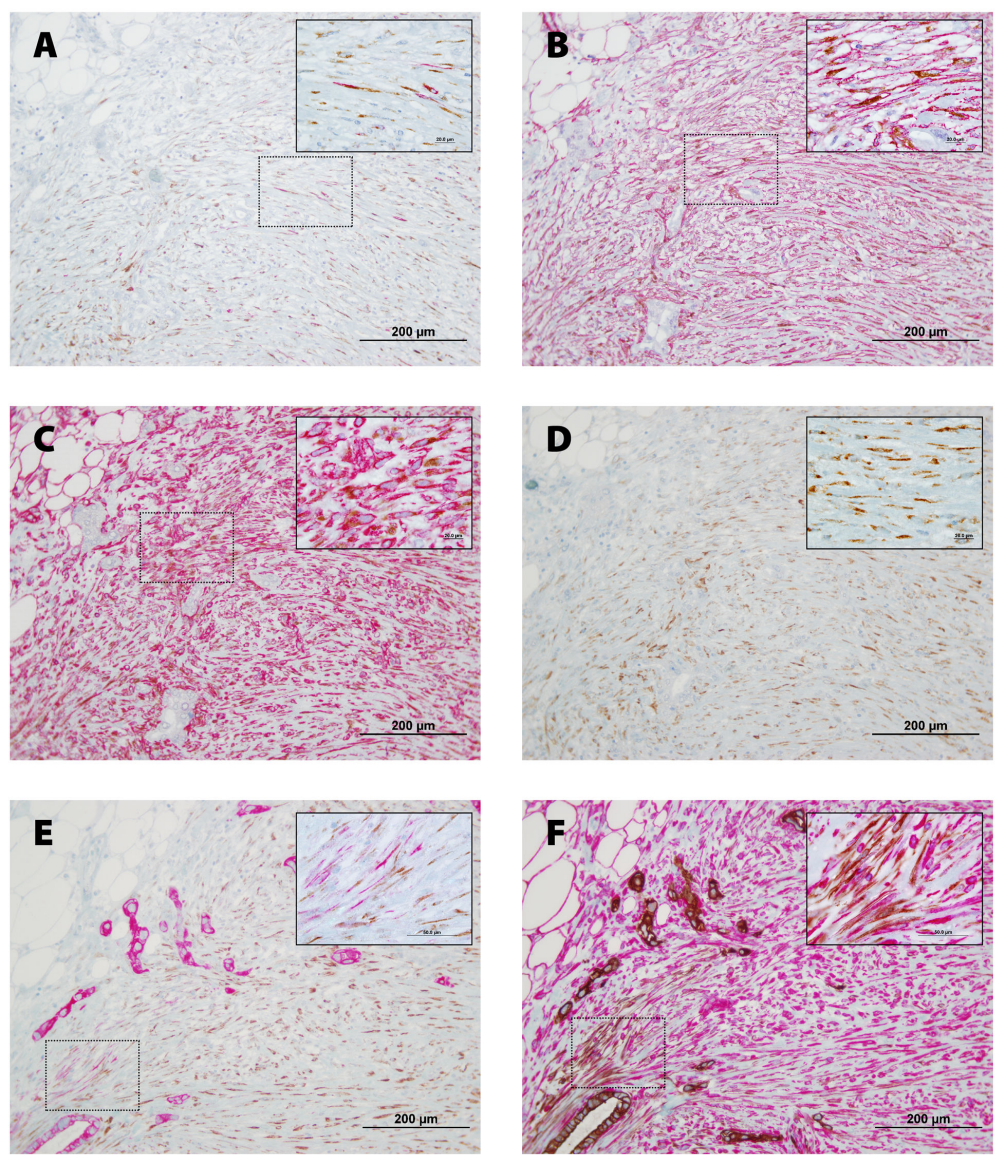
Co-staining of anti-proCOL11A1 mAb with different fibroblastic markers and CK 7, in Pancreatic Ductal Adenocarcinoma. **A**, anti-proCOL11A1 (brown) *vs*. desmin (magenta); **B**, anti-proCOL11A1 (brown) *vs*. αSMA (magenta); **C**, anti-proCOL11A1 (brown) *vs*. VIM (magenta); **D**, anti-proCOL11A1 (brown) *vs*. GFAP (not staining); **E**, anti-proCOL11A1 (brown) vs. CK7 (magenta); **F**, CK7 (epithelial tumor cells: *brown*) vs. VIM (magenta) (all photomicrographs at ×200, Scale bar 200 μm; inset X1000).

**Figure 4:**
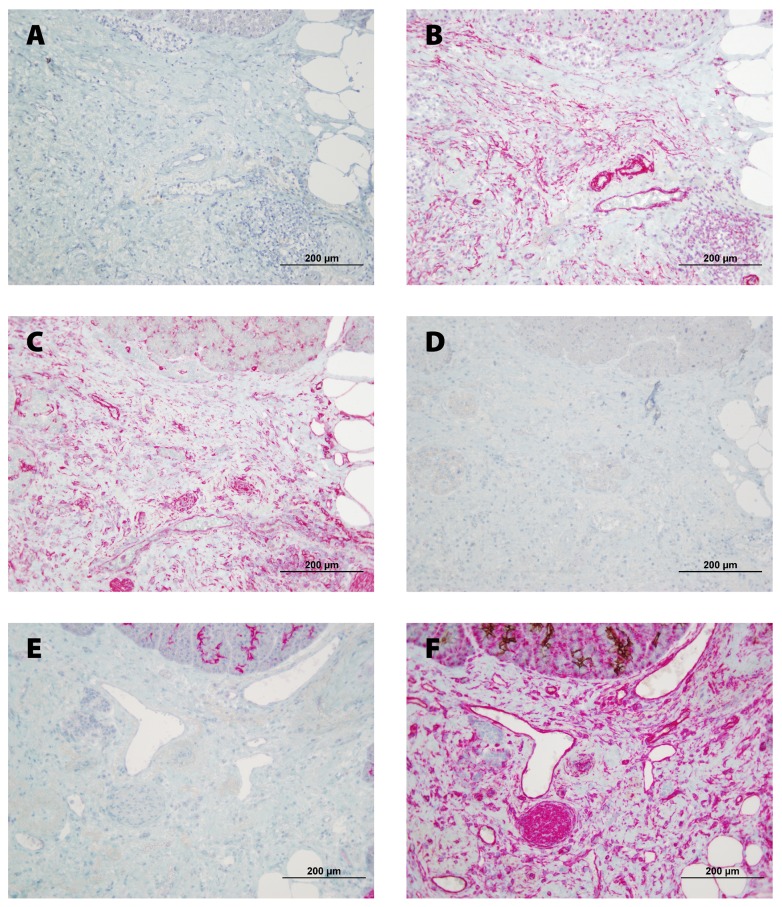
Co-staining of anti-proCOL11A1 mAb with different fibroblastic markers and CK 7, in Chronic Pancreatitis. **A**, anti-proCOL11A1 (brown) *vs*. desmin (magenta); **B**, Anti-proCOL11A1 (brown) *vs*. αSMA (magenta); **C**, anti-proCOL11A1 (brown) *vs*. VIM (magenta); **D**, anti-proCOL11A1 (brown) *vs*. GFAP (not staining); **E**, anti-proCOL11A1 (brown) vs. CK7 (magenta); **F**, CK7 (epithelial tumor cells: *brown*) vs. VIM (magenta).(all photomicrographs at ×200, Scale bar 200 μm). .

To characterize the CAFs, pancreatic tissue sections were double stained for proCOL11A1/desmin, proCOL11A1/αSMA, proCOL11A1/VIM, proCOL11A1/GFAP, proCOL11A1/CK7 and CK7/VIM (Figure 3). A very high number of mesenchymal cells were strongly labeled for VIM and αSMA. Immunostaining with GFAP was negative. As a proportion a small number of cells were proCOL11A1+ and desmin+. Co-staining of VIM or CK7 with proCOL11A1 identified a subset of CAFs with the mesenchymal phenotype (proCOL11A1+/VIM+) and very few cells with the epithelial phenotype (proCOL11A1+/CK7+) (Figures 3 and E, respectively). Some desmin or αSMA cells co-stained with proCOL11A1 (Figure 3 A and B, respectively). However, CP stroma samples did not show staining with proCOL11A1, desmin or GFAP, and there was no co-staining (Figure 4).

**Figure 5:**
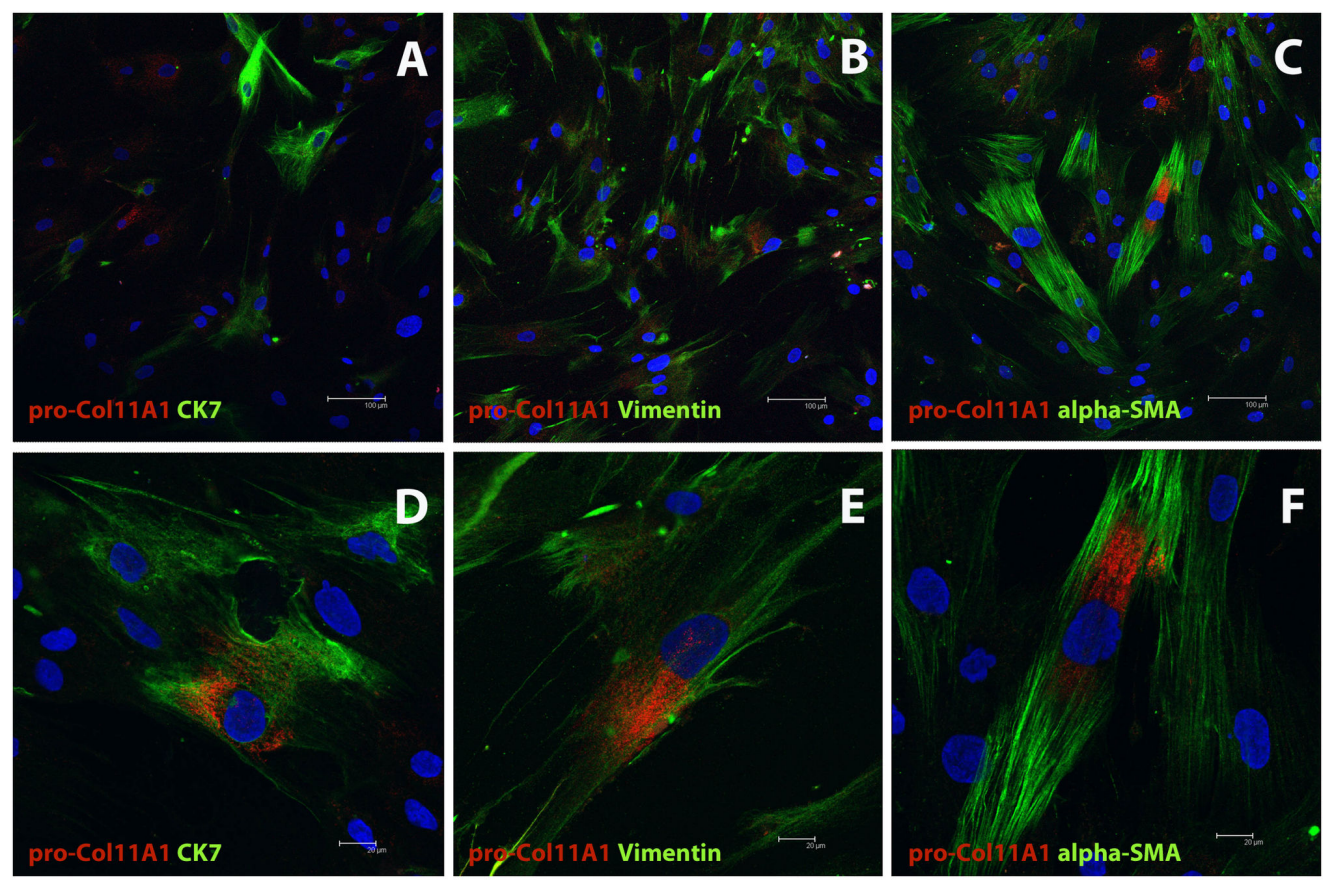
Confocal microscopy of pancreatic cultured CAFs. Double fluorescence stain illustrates the presence of cells proCOL11A1+/CK7+, proCOL11A1+/αSMA+ and proCOL11A1+/VIM+. *Red*: proCOL11A1; *green*: CK7, αSMA and VIM, respectively; *blue*: nuclei. Insets: randomly taken high power fields of culture. Scale bar 100 μm (X200) and 20 μm (X630).

The cell distribution of cultured CAFs was analyzed by colocalization with immunocytofluorescence. The resulting data are depicted in Table 2 (Figure 5). The interpretation of the table is as follows: when a double staining was applied to the cultured pancreatic CAFs, for instance proCOL11A1 and CK7, a total of 188 cells were stained; of these, 82 cells were labeled with anti-proCOL11A1, 60 cells were CK7 positive, and 46 cells were stained with both antibodies. The same logic applies to the rest of the stainings. According to the data shown in Table 2, among proCOL11A1 cells, 36% are CK7+, 49% are VIM+ and 48% are αSMA+; among the cells with the VIM phenotype, 21% are proCOL11A1+ and only 8% are CK7+; 33% of αSMA cells share the proCOL11A1 phenotype; and lastly, 45% of CK7 cells have the VIM+ phenotype and 43% have the proCOL11A1 phenotype. The distribution of the proCOL11A1, CK7 and desmin phenotypes in tissue samples is shown in Table S2 in File S1 . It was not possible to analyze those fields in which cells are VIM+ and αSMA+ due to the high number of stained cells, which makes it complicated to individualize and count them. According to the data shown in Table S2 in File S1, 18% of proCOl11A1 cells are CK7+, and 21% are desmin+; 45% of those cells staining with desmin have the proCOL11A1 phenotype, and 50% of CK7 cells are proCOL11A1.

**Table 2:** Quantitative analysis of cell distribution in cultured peritumoral pancreatic cancer fibroblasts (passage 3)*.

proCOL11A1/CK7 (DI)	proCOL11A1/VIM (DI)	proCOL11A1/αSMA (DI)	VIM/CK7 (DI)
proCOL11A1+ only 82 (43%)	proCOL11A1+ only 106 (18%)	proCOL11A1+ only 73 (23%)	VIM+ only 364 (85%)
CK7+ only 60 (32%)	VIM+ only 370 (64%)	αSMA+ only 138 (50%)	CK7+ only 36 (8%)
**proCOL11A1+/**CK7+ 46 (25%)	**proCOL11A1+/VIM+ 101 (18%)**	**proCOL11A1+/αSMA+ 67 (24%)**	**VIM+/CK7+ 30 (7%**)
Total 188 (100%)	Total 577 (100%)	Total 278 (100%)	Total 430 (100%)

* One patient sample, valuation on five fields for each double immunostaining (DI) experiment. Cells stained with both Ab in **bold.**

### PDAC vs. CP

**Figure 6:**
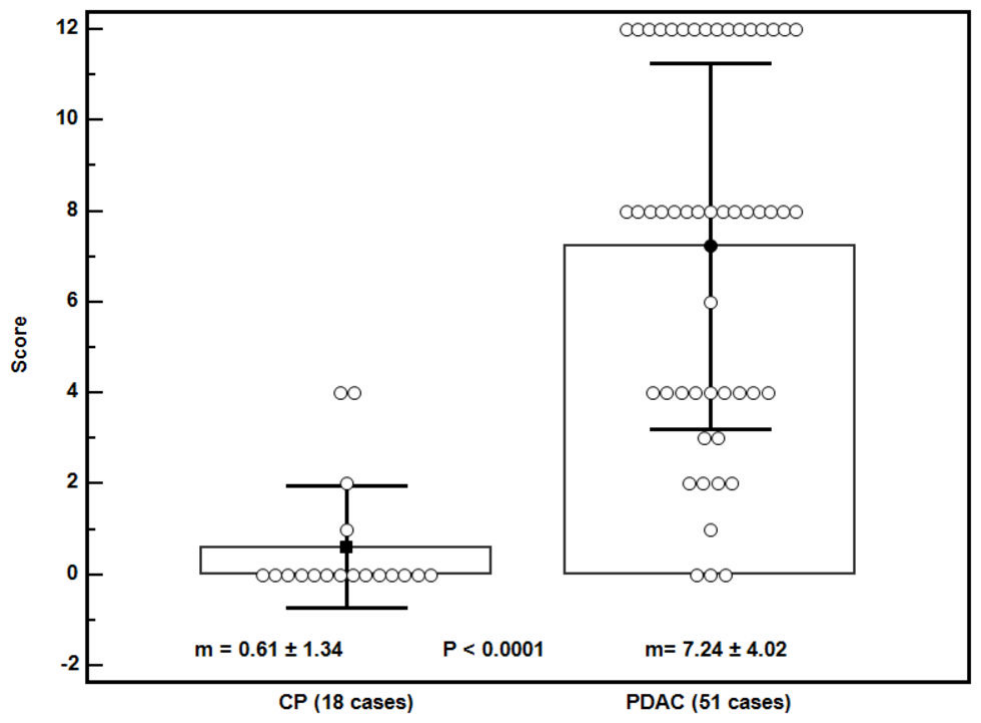
Plot of all data of immunostaining score with anti-proCOL11A1 mAb in chronic pancreatitis (CP) and pancreatic ductal adenocarcinoma (PDAC). Bars: ± 1 SD.

**Figure 7:**
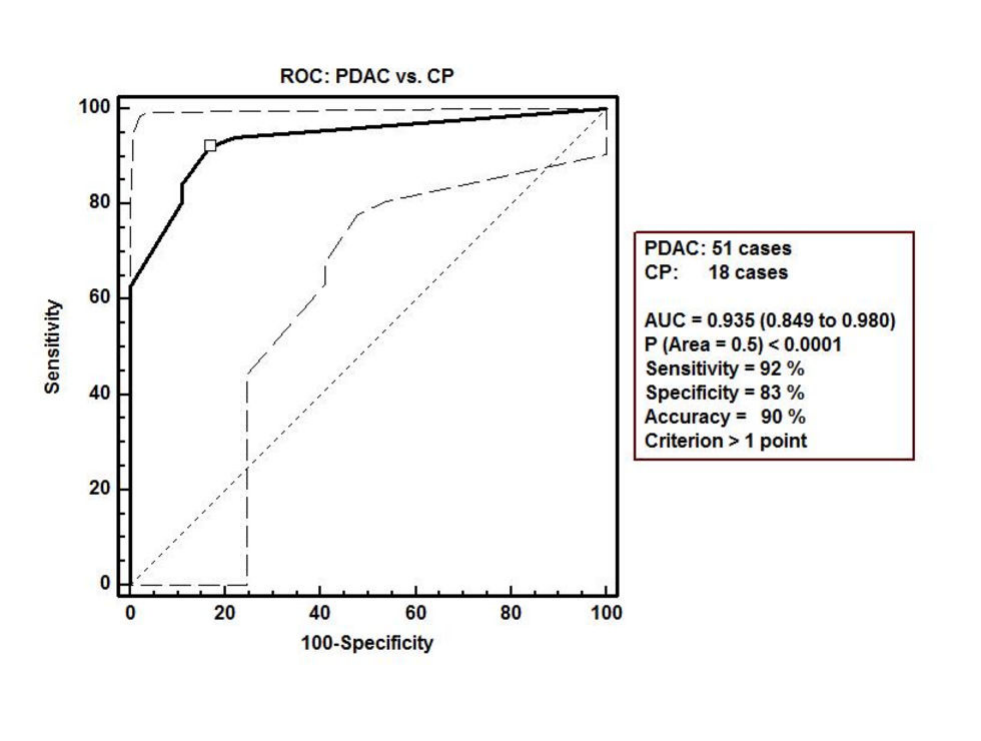
ROC curve with 95% confidence bounds of pancreatic ductal adenocarcinoma (PDAC) *vs.* chronic pancreatitis (CP) by immunostaining score with anti-proCOL11A1 mAb. µ indicates the cut-off point with the best separation (minimal false negative and false positive results) between the two groups.

The characteristics of each patient and his/her pathologist’s proCOL11A1 immunostaining score are shown in Table S3 in File S1. There was a statistically significant difference between PDAC and chronic pancreatitis in the mAb score (mean ± SD: 7.33 ± 4.04 vs. 0.61 ± 1.33; P < 0.0001). The data are shown in Figure 6. The AUC of ROC curves PDAC vs. CP was 0.936 (0.851 to 0.981), P < 0.0001 (Figure 7). The immunostaining showed a sensitivity of 92% and a specificity of 83% in discriminating PDAC from CP, with an accuracy of 90%. The use of pAb showed a similar outcome discriminating PDAC from CP (Table S4 in File S1). Observational concordance between pathologists was >90%. The staining score did not correlate to the patients’ age, sex, tumor stage or grade. The association with patient overall survival was not investigated because the number of PDAC patients with a low score was very low (4/51 cases).

Immunostaining was also evaluated using the QWin image analysis software. The results are shown in Table S5 in File S1. There is a statistical difference between PDAC and CP in the number of positive cells in the stained surface and in the reference area. Within the same series, there was a high concordance between the pathologist score and the QWin data such as the surface area of the stained cells and the number of positive cells (Spearman’s rank correlation coefficient = 0.825 and 0.825, respectively). ROC curve AUC data were obtained for the six image-analysis parameters (Table S6 in File S1). All parameters permit discrimination between PDAC and chronic pancreatitis. The most relevant parameters were the number of positive cells and the surface area of the stained cells.

## Discussion

DNA microarrays permit simultaneous analysis of the expression level of thousands of genes and could clarify the mechanisms of pancreatic carcinogenesis [26-31]. The first study to apply this technique to pancreatic cancer successfully identified a set of genes that is differentially expressed in pancreatic cancer [32]. Other studies led to the identification of both previously described and new candidate genes [33-39,50]. Most of the genes identified could not be validated by other more accurate techniques at either the mRNA or protein level. We have focused our studies on those genes with potential interest as markers and/or therapeutic targets [23], one of which is COL11A1.

In this report we verify the immunohistochemical expression of COL11A1 in PDAC. Previous observations on the expression of COL11A1 in other cancers [40-44] were limited to differential gene expression analyses validated by quantitative RT-PCR, Northern blots and/or in situ mRNA hybridization. Recently, the expression of collagen XI in both normal and malignant human colon tissue has been assessed by immunohistochemistry [45].

A computational analysis of gene expression in multiple cancers [46] reveals that overexpression of COL11A1 and other genes is a high-specificity biomarker for cancer invasion and predicts the response to neoadjuvant therapy. The downregulation of COL11A1 in in vitro co-cultures of pancreatic cancer and stromal cells [47] has been reported. These in vitro observations are not supported by ex vivo data; the artificial in vitro conditions did not fulfill all the requirements nor afford all the local factors conducive to high COL11A1 expression. 

To our knowledge, no specific marker for CAFs has been reported. Conventional staining did not show differences between CAFs and normal fibroblasts. Immunohistochemistry with VIM and αSMA are used to label fibroblast and activated fibroblasts, respectively, but vimentin is expressed in various cells, including endothelial cells, myoepithelial cells, glial cells, and cells of the hematopoietic lineages. Likewise alpha αSMA expression can be found in pericytes, vascular smooth muscle cells and myoepithelial cells. Kalluri [48] has shown that CAFs are a heterogeneous population, and the use of VIM or αSMA as the only markers will not identify all of the CAFs, because there is a cellular overlap between these markers. They also stain cells that are involved in biological processes not associated with cancer, such as cells involved in epithelial-mesenchymal transition (EMT).

Few data exist about the number of cells with the EMT phenotype in cancer stroma. Raviraj [49] estimated that 2 cells with the EMT phenotype (VIM+/CK+) were present in matrices near a cluster of 30 breast tumoral cells. Our studies with double staining in pancreatic samples and double immunolabeling in cultured stromal cells also demonstrate that pancreatic CAFs are a heterogeneous population, and that some proCOL11A1 stromal cells co-stain with VIM, αSMA, desmin and a few cells with CK7. We tried to quantify the different CAF populations using immunocytofluorescence of cultured stromal cells obtained from surgical samples (Table 2). Although cultured stromal cells are not as representative as tissue cells, the data obtained could give a first approximation about the distribution of the CAF subpopulations. We found that the percentage of cells with the EMT phenotype (VIM+/CK7+) in our cultured CAFs was 7%, which could be considered close to reality. Interestingly, the percentage of cultured CAFs with the proCOL11A17/CK7 phenotype was 25%. Our histological studies show that there are some CAFs with the proCOL11A1/CK7 phenotype, and that epithelial cells do not stain with anti-proCOL11A1. All of this suggests that a fraction of the proCOL11A1+ stromal cells could participate in the EMT. Work is in progress in order to confirm this hypothesis. Another reading of Table 2 is that the populations of VIM+ cells and αSMA+ were very numerous, and not all these cells can be considered CAFs, but rather as another kind of stromal cells that do not participate in the progression of the tumor. We think that the CAF designation should be applied to a subset of fibroblast-like stromal cells in the peritumoral region that influence cancer growth and are identified by the anti-proCOL11A1 antibody.

Erkan et al. [50], applying DNA arrays, found that COL11A1 was highly pancreas-specific. They used αSMA and a commercial COL11A1 antibody to evaluate the co-localization, concluding that COL11A1 is a specific marker for pancreatic stellate cells (PSCs), but we observe that the number of such COL11A1+ cells was too numerous for all of them to have been PSCs. On the other hand, we cannot stain PSCs with GFAP but with desmin, and those desmin positive cells co-stain with anti-proCOL11A1 (Figure 2F; Figure 3D). Furthermore, the commercial antibody he employed also stained pancreatic acini, hepatocytes and chronic pancreatitis fibroblasts. In our experience, commercial antibodies other than anti-proCOL11A1 fail to stain desmoplastic stroma and show unspecific staining of cancer cells and benign tissue. The antibody anti-proCOL11A1 that we generated could be considered specific to pancreatic CAFs, and co-localization with the PSC marker desmin showed that only a subset of proCOL11A1+ cells could be considered PSCs. We have applied the antibody against human pro-COL11A1 to breast, colon, neck and head tumors [51-53] (papers in preparation), and the outcome is very similar to pancreatic cancer: it almost exclusively stains CAFs. Our current data highlight the role of stroma in pancreatic cancer biology. The present cancer paradigm is that tumor growth is not just determined by malignant cells, but also by tumor stroma [54,55]. The focal pattern of COL11A1 staining is restricted to defined areas of PDAC/CAF interaction while absent in chronic pancreatitis, which suggests that its expression is highly dependent on local factors coming from the neighboring carcinoma cells. In that sense, COL11A1 and CK7 co-expression in the same cell could either mean that the cell is a tumoral epithelial cell in the process of EMT (with the double epithelial/mesenchymal phenotype) or that the cell is a mesenchymal stem cell presenting with the double phenotype. To this effect, our team showed in previous reports [56,57] that the association of Capan-1 cells with CAFs results in a very aggressive orthotopic human pancreatic carcinoma xenograft model, with aggressive local tumor growth and the presence of metastasis.

Our analysis has shown that proCOL11A1 presents a strong immunohistochemical staining within the desmoplastic stroma of PDAC, but has a null or very weak expression in chronic pancreatitis. Moreover, proCOL11A1 expression could be seen in microscopic fields where tumor cells were not detected, an observation that highlights the usefulness of this marker in helping pathologists differentiate PDAC from CP. Both conditions are characterized by inflammatory events. On the basis of sample biopsies alone, and in clinical settings, it can be difficult to distinguish pancreatic cancer from chronic pancreatitis, and the frequent association of chronic pancreatitis with pancreatic cancer causes additional diagnostic problems. Immunohistochemical staining has been used to differentiate pancreatic adenocarcinoma from chronic pancreatitis (Table S7 in File S1). In general, the higher sensitivity and specificity values obtained in most cases were with small sample sizes and weak power tests (high type II error), and therefore, the differentiation of pancreatic carcinoma from chronic pancreatitis with those immunostaining markers would not be statistically significant. The sample size used in our immunohistochemical study (n=78) allowed us to achieve statistical significance with a statistical power of 90%. The pathologist score of the anti-proCOL11A1 immunostaining also allows classification of the patients with 90% accuracy; that is, it makes it easier to distinguish between chronic pancreatitis and PDAC in clinical settings. Our score has two components: number of positive fields (0-4) and the field’s staining intensity (0-3), giving a total score of 0-12. Both components can individually already discriminate between PDAC and CP, using the criterion of more than 1 positive field, or ≥ 1% of positive cells in relation to the stromal surface (Table S8 in File S1). We reviewed the false positive/negative cases with the monoclonal antibody. There were three PDAC cases with zero points (Table S3 in File S1: cases 41, 44 and 54); one was a case whose diagnosis was not ultimately established with accuracy (distal common bile duct cancer vs. PDAC). The maximum score in CP patients was 4 points in two patients, one of them suffering from autoimmune pancreatitis (AIP) (Table S3 in File S1: cases 15 and 31, Figure S3 in File S1). Its preoperative usefulness with core needle biopsy, as we demonstrated in breast cancer [51,52], needs to be investigated in pancreatic cancer. Whether proCOL11A1 can be useful as a differential diagnosis marker using non-invasive techniques must be explored as well. The significance of the expression of pro-Col11A1 in our AIP case should be explored in view of its possible relationship with the particular composition of collagens in the AIP stroma. If this finding is confirmed by further studies, it could add data that will contribute to an improved understanding of the pathogenesis of this disease and the development of new diagnostic biomarkers for AIP.

Our gene expression data and the subsequent immunohistochemical validation stress that the differential expression of COL11A1 has diagnostic significance, as it allows for distinction between CP and PDAC. We have generated a highly specific human proCOL11A1- antibody that can be applied in order to immunoscreen the expression of the protein in human tissues. In investigations on CAFs, it is fundamental to determine which stromal cells are truly cancer-specific and which ones are non-cancer-specific activated fibroblasts. In this field, the application of anti-proCOL11A1 could be useful. Our work shows that PDAC stromal fibroblasts stain with anti-proCOL11A1. They have the morphological characteristics of myofibroblasts and a subgroup thereof could be stellate cells. Our data suggest that cells that express proCOL11A1 could participate in EMT. The fact that the staining with anti-proCOL11A1 is negative in normal pancreas stromal cells and almost absent in a benign inflammatory process like CP suggests that this staining could be a specific marker for CAFs. Therefore, anti-proCOL11A1 can be seen to be a significant marker of high diagnostic and clinical relevance.

## Supporting Information

File S1 **Supporting Figures and Tables**.
Figure S1, Immunohistochemical staining with anti-proCOL11A1 pAb of Chronic Pancreatitis (CP) and Pancreatic Ductal Adenocarcinoma (PDAC) . A: CP (H&E). B: CP negative anti-proCOL11A1 stain. C: PDAC (H&E). D: PDAC positive anti-proCOL11A1 stain. E and F: Detail of anti-proCOL11A1 expression in stromal cells of PDAC. H & E indicates Hematoxilin and Eosin (all photomicrographs at ×400). Figure S2, Immunohistochemical staining of normal pancreas with different fibroblastic markers. A, anti-proCOL11A1 mAb, positive control (inset): cell line A204; B, desmin, positive control (inset): appendix; C, alpha-Smooth Muscle Actin, positive control (inset): appendix; D, vimentin, positive control (inset) : appendix) ; E, GFAP, positive control (inset) : astrocytoma). Serial sections (all photomicrographs at ×200, Scale bar 200 μm). Figure S3, Immunohistochemical staining of autoimmune pancreatitis with different fibroblastic markers. A, anti-proCOL11A1 mAb , positive control (inset): cell line A204); B, desmin, positive control (inset): appendix); C, alpha-Smooth Muscle Actin , positive control (inset) : appendix) ; D, vimentin , positive control (inset): appendix) ; E, GFAP, positive control (inset) : astrocytoma). Serial sections (all photomicrographs at ×200, Scale bar 200 μm). Table S1, Comparison of gene expression data from microarray analysis (Affymetrix GeneChips). Table S2, Quantitative analysis of cell distribution in peritumoral pancreatic cancer tissue. Table S3, Patient characteristics and immunohistochemistry score with anti-proCOL11A1 pAb and mAb. Table S4, Discrimination between PDAC (pancreatic ductal adenocarcinoma) and CP (chronic pancreatitis) using pathologist score. Table S5, Summary statistics of immunohistochemical analyses. Table S6, Area under the ROC curve (AUC) of image analysis parameters (pancreatic ductal adenocarcinoma) PDAC vs. CP (chronic pancreatitis). Table S7, Discrimination between PDAC (pancreatic ductal adenocarcinoma) and CP (chronic pancreatitis) using various diagnostic markers in tissues. Table S8, Discrimination PDAC (pancreatic ductal adenocarcinoma) vs. CP (chronic pancreatitis) with anti-proCOL11A1 mAb by components of score and total score.
(DOCX)

File S2 **Global Gene Expression Analysis.** Generation of a rabbit polyclonal antiserum to the variable region of human procollagen 11A1 (anti-proCOL11A1 pAb). SDS-PAGE and Western-blots.
(DOCX)
