# Supplementary material for: Overexpression of COL11A1 by Cancer-Associated Fibroblasts: Clinical Relevance of a Stromal Marker in Pancreatic Cancer
Source: PLoS One. 2013 Oct 23;8(10):e78327. doi: 10.1371/journal.pone.0078327 (PMC3808536; doi:10.1371/journal.pone.0078327)
Supplement: File S1 — Supporting Figures and Tables. Figure S1, Immunohistochemical staining with anti-proCOL11A1 pAb of Chronic Pancreatitis (CP) and Pancreatic Ductal Adenocarcinoma (PDAC) . A: CP (H&E). B: CP negative anti-proCOL11A1 stain. C: PDAC (H&E). D: PDAC positive anti-proCOL11A1 stain. E and F: Detail of anti-proCOL11A1 expression in stromal cells of PDAC. H & E indicates Hematoxilin and Eosin (all photomicrographs at ×400). Figure S2, Immunohistochemical staining of normal pancreas with different fibroblastic markers. A, anti-proCOL11A1 mAb, positive control (inset): cell line A204; B, desmin, positive control (inset): appendix; C, alpha-Smooth Muscle Actin, positive control (inset): appendix; D, vimentin, positive control (inset) : appendix) ; E, GFAP, positive control (inset) : astrocytoma). Serial sections (all photomicrographs at ×200, Scale bar 200 μm). Figure S3, Immunohistochemical staining of autoimmune pancreatitis with different fibroblastic markers. A, anti-proCOL11A1 mAb , positive control (inset): cell line A204); B, desmin, positive control (inset): appendix); C, alpha-Smooth Muscle Actin , positive control (inset) : appendix) ; D, vimentin , positive control (inset): appendix) ; E, GFAP, positive control (inset) : astrocytoma). Serial sections (all photomicrographs at ×200, Scale bar 200 μm). Table S1, Comparison of gene expression data from microarray analysis (Affymetrix GeneChips). Table S2, Quantitative analysis of cell distribution in peritumoral pancreatic cancer tissue. Table S3, Patient characteristics and immunohistochemistry score with anti-proCOL11A1 pAb and mAb. Table S4, Discrimination between PDAC (pancreatic ductal adenocarcinoma) and CP (chronic pancreatitis) using pathologist score. Table S5, Summary statistics of immunohistochemical analyses. Table S6, Area under the ROC curve (AUC) of image analysis parameters (pancreatic ductal adenocarcinoma) PDAC vs. CP (chronic pancreatitis). Table S7, Discrimination between PDAC (pancreatic ductal adeno [file pone.0078327.s001.docx]

**Table S1**. **Gene expression data from microarray analysis (Affymetrix chips) of pancreatic samples. Genes with highest overexpression between PDAC and chronic pancreatitis/normal pancreas**

| **Affymetrix probe set ID** | **Gene description** | **Gene symbol** | **Gene ID** | **P value** |
| --- | --- | --- | --- | --- |
| 202381_at | ADAM metallopeptidase domain 9 | ADAM9 | NM_003816 | 0,04180 |
| 202207_at | ADP-ribosylation factor-like 7 | ARL7 | BG435404 | 0,00423 |
| 206561_s_at | aldo-keto reductase family 1, member B10 | AKR1B10 | NM_020299 | 0,05050 |
| 203559_s_at | amiloride binding protein 1 | ABP1 | NM_001091 | 0,01560 |
| 209173_at | anterior gradient 2 homolog | AGR2 | AF088867 | 0,00348 |
| 204416_x_at | apolipoprotein C-I | APOC1 | NM_001645 | 0,01070 |
| 203382_s_at | apolipoprotein E | APOE | NM_000041 | 0,00972 |
| 209281_s_at | ATPase, Ca++ transporting, plasma membrane 1 | ATP2B1 | L14561 | 0,30000 |
| 207173_x_at | cadherin 11, type 2, OB-cadherin (osteoblast) | CDH11 | D21254 | 0,00293 |
| 201884_at | carcinoembryonic antigen cell adhesion molecule 5 | CEACAM5 | NM_004363 | 0,00114 |
| 203757_s_at | carcinoembryonic antigen cell adhesion molecule 6 | CEACAM6 | BC005008 | 0,24500 |
| 205713_s_at | cartilage oligomeric matrix protein | COMP | NM_000095 | 0,00241 |
| 200838_at | cathepsin B | CTSB | NM_001908 | 0,01240 |
| 205927_s_at | cathepsin E | CTSE | NM_001910 | 0,00303 |
| 205173_x_at | CD58 antigen | CD58 | NM_001779 | 0,02710 |
| 201005_at | CD9 antigen (p24) | CD9 | NM_001769 | 0,01850 |
| 204170_s_at | CDC28 protein kinase regulatory subunit 2 | CKS2 | NM_001827 | 0,00307 |
| 210559_s_at | cell division cycle 2, G1 to S and G2 to M | CDC2 | D88357 | 0,00597 |
| 209201_x_at | chemokine (C-X-C motif) receptor 4 | CXCR4 | L01639 | 0,00003 |
| 204619_s_at | chondroitin sulfate proteoglycan 2 | CSPG2 | BF590263 | 0,00013 |
| 201310_s_at | chromosome 5 open reading frame 13 | C5orf13 | NM_004772 | 0,00023 |
| 206284_x_at | clathrin, light polypeptide (Lcb) | CLTB | NM_001834 | 0,02960 |
| 202404_s_at | collagen, type I, alpha 2 | COL1A2 | NM_000089 | 0,01010 |
| 201852_x_at | collagen, type III, alpha 1 | COL3A1 | NM_000090 | 0,02140 |
| 212489_at | collagen, type V, alpha 1 | COL5A1 | AI983428 | 0,00004 |
| 217428_s_at | collagen, type X, alpha 1 | COL10A1 | X98568 | 0,00070 |
| **37892_at** | **collagen, type XI, alpha 1** | **COL11A1** | **J04177** | **0,00008** |
| 204636_at | collagen, type XVII, alpha 1 | COL17A1 | NM_000494 | 0,17700 |
| 205081_at | cysteine-rich protein 1 (intestinal) | CRIP1 | NM_001311 | 0,00229 |
| 205765_at | cytochrome P450, family 3, subfamily A, ppe 5 | CYP3A5 | NM_000777 | 0,05110 |
| 206439_at | dermatan sulfate proteoglycan 3 | DSPG3 | NM_004950 | 0,01800 |
| 206414_s_at | development and differentiation enhancing factor 2 | DDEF2 | NM_003887 | 0,00556 |
| 201430_s_at | dihydropyrimidinase-like 3 | DPYSL3 | W72516 | 0,00342 |
| 201697_s_at | DNA (cytosine-5-)-methyltransferase 1 | DNMT1 | NM_001379 | 0,06830 |
| 31845_at | E74-like factor 4 (ets domain transcription factor) | ELF4 | U32645 | 0,01130 |
| 209682_at | ecotropic retroviral transforming sequence b | CBLB | U26710 | 0,00059 |
| 204858_s_at | endothelial cell growth factor 1 | ECGF1 | NM_001953 | 0,04460 |
| 204464_s_at | endothelin receptor type A | EDNRA | NM_001957 | 0,00072 |
| 201231_s_at | enolase 1, (alpha) | ENO1 | NM_001428 | 0,00847 |
| 202668_at | ephrin-B2 | EFNB2 | BF001670 | 0,00600 |
| 202609_at | epidermal growth factor receptor, substrate 8 | EPS8 | NM_004447 | 0,06910 |
| 219787_s_at | epithelial cell transforming sequence 2 oncogene | ECT2 | NM_018098 | 0,01400 |
| 205419_at | Epstein-Barr virus induced gene 2 | EBI2 | NM_004951 | 0,00089 |
| 216836_s_at | erythroblastic leukemia viral oncogene homolog 2 | ERBB2 | X03363 | 0,08650 |
| 201798_s_at | fer-1-like 3, myoferlin (C. elegans) | FER1L3 | NM_013451 | 0,01770 |
| 209955_s_at | fibroblast activation protein, alpha | FAP | U76833 | 0,00214 |
| 212464_s_at | fibronectin 1 | FN1 | X02761 | 0,00330 |
| 202949_s_at | four and a half LIM domains 2 | FHL2 | NM_001450 | 0,14200 |
| 201141_at | glycoprotein (transmembrane) nmb | GPNMB | NM_002510 | 0,01960 |
| 217771_at | golgi phosphoprotein 2 | GOLPH2 | NM_016548 | 0,21700 |
| 218468_s_at | gremlin 1, cysteine knot superfamily | GREM1 | NM_013372 | 0,07060 |
| 202581_at | heat shock 70kDa protein 1B | HSPA1B | NM_005346 | 0,03870 |
| 218280_x_at | histone 2, H2aa | HIST2H2 | BC001629 | 0,02070 |
| 213299_at | HIV-1 inducer of short transcripts binding protein | FBI1 | NM_015898 | 0,11200 |
| 211597_s_at | homeodomain-only protein | HOP | AB059408 | 0,00732 |
| 211430_s_at | immunoglobulin heavy constant mu | IGHM | M87789 | 0,00001 |
| 214677_x_at | immunoglobulin lambda locus | IGLJ3 | X57812 | 0,00256 |
| 210511_s_at | inhibin, beta A | INHBA | M13436 | 0,00007 |
| 210095_s_at | insulin-like growth factor binding protein 3 | IGFBP3 | M31159 | 0,00004 |
| 206502_s_at | insulinoma-associated 1 | INSM1 | NM_002196 | 0,21900 |
| 214660_at | integrin, alpha subunit | ITGA1 | X68742 | 0,03400 |
| 202859_x_at | interleukin 8 | IL8 | NM_000584 | 0,01600 |
| 205157_s_at | keratin 17 | KRT17 | NM_000422 | 0,00585 |
| 201650_at | keratin 19 | KRT19 | NM_002276 | 0,12400 |
| 203726_s_at | laminin, alpha 3 | LAMA3 | NM_000227 | 0,00513 |
| 209270_at | laminin, beta 3 | LAMB3 | L25541 | 0,01490 |
| 201105_at | lectin, galactoside-binding, soluble, 1 | LGALS1 | NM_002305 | 0,04540 |
| 208949_s_at | lectin, galactoside-binding, soluble, 3 (galectin 3) | LGALS3 | BC001120 | 0,06000 |
| 221558_s_at | lymphoid enhancer-binding factor 1 | LEF1 | AF288571 | 0,00008 |
| 209480_at | Major histocompatibility complex, class II | HLA | M16276 | 0,00345 |
| 209373_at | mal, T-cell differentiation protein-like | MALL | BC003179 | 0,02550 |
| 204475_at | matrix metallopeptidase 1 | MMP1 | NM_002421 | 0,77600 |
| 204580_at | matrix metallopeptidase 12 | MMP12 | NM_002426 | 0,00147 |
| 204885_s_at | mesothelin | MSLN | NM_005823 | 0,01830 |
| 209035_at | midkine (neurite growth-promoting factor 2) | MDK | M69148 | 0,00223 |
| 202555_s_at | myosin, light polypeptide kinase | MLCK | NM_005965 | 0,21000 |
| 201621_at | neuroblastoma, suppression of tumorigenicity 1 | NBL1 | NM_005380 | 0,00751 |
| 209627_s_at | oxysterol binding protein-like 3 | OSBPL3 | AY008372 | 0,03880 |
| 210809_s_at | periostin, osteoblast specific factor | POSTN | D13665 | 0,00280 |
| 201037_at | phosphofructokinase, platelet | PFKP | NM_002627 | 0,00026 |
| 203554_x_at | pituitary tumor-transforming 1 | PTTG1 | NM_004219 | 0,00005 |
| 205190_at | plastin 1 (I isoform) | PLS1 | NM_002670 | 0,07220 |
| 218644_at | pleckstrin 2 | PLEK2 | NM_016445 | 0,00634 |
| 209803_s_at | pleckstrin homology-like domain, family A, m 2 | PHLDA2 | AF001294 | 0,19900 |
| 201251_at | pyruvate kinase, muscle | PKM2 | NM_002654 | 0,00813 |
| 217763_s_at | RAB31, member RAS oncogene family | RAB31 | AF183421 | 0,01540 |
| 218657_at | Rap guanine nucleotide exchange factor -like 1 | RGEFL1 | NM_016339 | 0,01760 |
| 202988_s_at | regulator of G-protein signalling 1 | RGS1 | NM_002922 | 0,00658 |
| 212724_at | Rho family GTPase 3 | RND3 | BG054844 | 0,05240 |
| 201288_at | Rho GDP dissociation inhibitor (GDI) beta | ARHGDIB | NM_001175 | 0,01750 |
| 201890_at | ribonucleotide reductase M2 polypeptide | RRM2 | BE966236 | 0,00575 |
| 209360_s_at | runt-related transcription factor 1 | RUNX1 | BF432501 | 0,01530 |
| 200872_at | S100 calcium binding protein A10 | S100A10 | NM_002966 | 0,00402 |
| 200660_at | S100 calcium binding protein A11 | S100A11 | NM_005620 | 0,06420 |
| 204351_at | S100 calcium binding protein P | S100P | NM_005980 | 0,00220 |
| 200665_s_at | secreted protein, acidic, cysteine-rich | SPARC | NM_003118 | 0,00421 |
| 203789_s_at | sema domain, immunoglobulin domain | SEMA3C | NM_006379 | 0,00528 |
| 204855_at | serpin peptidase inhibitor, clade B, member 5 | SERPINB5 | NM_002639 | 0,00434 |
| 202628_s_at | serpin peptidase inhibitor, clade E | SERPINE1 | NM_000602 | 0,00689 |
| 209016_s_at | Similar to keratin 7 | KRT7 | BC002700 | 0,00230 |
| 204401_at | small conductance calcium-activated channel | KCNN4 | NM_002250 | 0,05010 |
| 202856_s_at | solute carrier family 16 | SLC16A3 | NM_004207 | 0,00245 |
| 204588_s_at | solute carrier family 7 | SLC7A7 | NM_003982 | 0,00019 |
| 33322_i_at | stratifin | SFN | X57348 | 0,05650 |
| 203083_at | thrombospondin 2 | THBS2 | NM_003247 | 0,00002 |
| 201666_at | TIMP metallopeptidase inhibitor 1 | TIMP1 | NM_003254 | 0,03270 |
| 201291_s_at | topoisomerase (DNA) II alpha 170kDa | TOP2A | AU159942 | 0,20900 |
| 201506_at | transforming growth factor, beta-induced | TGFBI | NM_000358 | 0,00719 |
| 201042_at | transglutaminase 2 | TGM2 | AL031651 | 0,12600 |
| 218960_at | transmembrane protease, serine 4 | TMPRSS4 | NM_016425 | 0,00544 |
| 214476_at | trefoil factor 2 (spasmolytic protein 1) | TFF2 | NM_005423 | 0,26200 |
| 202504_at | tripartite motif-containing 29 | TRIM29 | NM_012101 | 0,00968 |
| 204083_s_at | tropomyosin 2 (beta) | TPM2 | NM_003289 | 0,31500 |
| 215111_s_at | TSC22 domain family, member 1 | TSC22D1 | AK027071 | 0,03870 |
| 208623_s_at | villin 2 (ezrin) | VIL2 | J05021 | 0,18800 |
| 209950_s_at | villin-like | VILL | BC004300 | 0,06840 |

List of genes overexpressed in PDAC compared to controls (normal pancreas and chronic pancreatitis) based on Affymetrix GCOS 1.2 software. **Bold**: COL11A1 gene Additionally, the p-value of the overexpression based on a parametric Welch t-test is also indicated (GeneSpring; Silicon Genetics, Redwood City, CA).

**Table S2. Quantitative analysis of cell distribution in peritumoral pancreatic cancer tissue.**

| proCOL11A1/CK7 (DI) | proCOL11A1/Desmin (DI) |
| --- | --- |
| proCOL11A1+ only 123 (60%) | proCOL11A1+ only 140 (63%) |
| CK7+ only 28 (15%) | Desmin+ only 45 (20%) |
| **proCOL11A1+/CK7+ 28 (15%)** | **proCOL11A1+/Desmin+ 37 (17%)** |
| Total 119 (100%) | Total 222 (100%) |

One patient sample, valuation on four fields for each double immunostaining (DI) experiment. Cells stained with both Ab in **bold**

**Table S3. Patient characteristics and immunohistochemistry score with anti-proCOL11A1 pAb and mAb.**

| PATIENT | SEX | AGE | pAb-Score | mAb-Score | TNM | STAGE | GRADE | PATHOLOGY |
| --- | --- | --- | --- | --- | --- | --- | --- | --- |
| 1 | F | 74 | 12 | 4 | T3N0M0 | IIA | G2 | PDAC |
| 2 | F | 71 | 12 | 4 | T1N0M0 | IA | G1 | PDAC |
| 3 | M | 54 | 4 | 2 | T2NOM0 | IB | G2 | PDAC |
| 4 | F | 46 | 8 | 8 | T2N1M1 | IV | G2-3 | PDAC |
| 5 | M | 72 | 12 | 2 | T2N0M0 | IB | G2 | PDAC |
| 6 | M | 36 | 0 | 1 |  |  |  | CP |
| 7 | M | 69 | 8 | 4 | T3N0M0 | IIA | G2 | PDAC |
| 8 | M | 71 | 0 | 8 | T1N0M0 | IA | G2 | PDAC |
| 9 | F | 77 | 12 | 12 | T3N0M0 | IIA | G2 | PDAC |
| 10 | M | 73 | 12 | 12 | T3N0M0 | IIA | G2 | PDAC |
| 11 | M | 65 | 0 |  |  |  |  | CP |
| 12 | F | 63 | 12 | 8 | T3N1M0 | IIB | G3 | PDAC |
| 13 | F | 81 | 8 | 8 | T2N0M0 | IB | G2 | PDAC |
| 14 | M | 52 | 0 | 2 |  |  |  | CP |
| ***15*** | ***M*** | ***63*** | ***0*** | ***4*** |  |  |  | ***CP*** |
| 16 | M | 51 | 8 | 3 | T3N1M0 | IIB | G2 | PDAC |
| 17 | F | 52 | 0 | 0 |  |  |  | CP |
| 18 | M | 48 | 0 |  |  |  |  | CP |
| 19 | M | 73 | 0 |  |  |  |  | CP |
| 20 | M | 52 | 0 | 0 |  |  |  | CP |
| 21 | M | 59 | 0 | 0 |  |  |  | CP |
| 22 | M | 74 | 12 |  | T1N0M0 | IA | G2 | PDAC |
| 23 | F | 64 | 12 | 8 | T3N0M0 | IIA | G2 | PDAC |
| 24 | M | 56 | 0 | 0 |  |  |  | CP |
| 25 | M | 44 | 0 | 0 |  |  |  | CP |
| 26 | M | 55 | 12 | 12 | T2NOMO | IA | G1 | PDAC |
| 27 | F | 57 | 4 | 2 | T2N1M0 | IIB | G2 | PDAC |
| 28 | M | 61 | 0 | 0 |  |  |  | CP |
| 29 | M | 61 | 0 | 0 |  |  |  | CP biliar |
| 30 | M | 60 | 8 | 8 | T4N1M0 | III | G1-2 | PDAC |
| ***31*** | ***M*** | ***73*** | ***8*** | ***4*** |  |  |  | ***autoimmune pancreatitis*** |
| 32 | F | 55 | 12 | 12 | T3N1M0 | IIB | G2 | PDAC |
| 33 | M | 56 | 8 | 0 |  |  |  | CP |
| 34 | M | 70 | 12 | 4 | T3N1M0 | IIB | G2 | PDAC |
| 35 | F | 49 | 4 | 6 | T3N0M1 | IV | G1 | PDAC |
| 36* | F | 71 | 12 | 12 | TXNXM1 | IV |  | PDAC |
| 37 | F | 69 | 8 | 12 | T3N1M0 | IIB |  | PDAC |
| 38 | M | 65 | 8 | 4 | T3N1M0 | IIB | G1 | PDAC |
| 39 | M | 49 | 0 |  |  |  |  | CP |
| 40* | M | 60 | 3 |  | T4N1M0 | IIB |  | PDAC |

Table S1 *(Cont.)*

| PATIENT | SEX | AGE | pAb-Score | mAb-Score | TNM | STAGE | GRADE | PATHOLOGY |
| --- | --- | --- | --- | --- | --- | --- | --- | --- |
| **41** | **F** | **64** | **0** | **0** | **T1N0MO** | **IA** | **G2** | **PDAC** |
| 42 | F | 53 | 12 | 12 | T3N0M0 | IIA | G3 | PDAC |
| 43 | M | 69 | 0 | 0 |  |  |  | CP |
| **44** | **M** | **73** | **2** | **0** | **T2N0M0** | **IB** | **G2** | **PDAC** |
| 45 | M | 53 | 0 |  |  |  |  | CP |
| 46 | F | 66 | 4 | 4 | T3N1M0 | IIB | G1 | PDAC |
| 47 | M | 62 | 12 | 8 | T3N1M0 | IIB | G1 | PDAC |
| 48 | F | 78 | 12 | 12 | T2N0M0 | IB | G2 | PDAC |
| 49 | M | 77 | 12 | 3 | T3N0M0 | IIA | G2 | PDAC |
| 50 | M | 62 | 4 | 12 | T2N0M0 | IB | G1 | PDAC |
| 51 | M | 41 | 0 | 0 |  |  |  | CP |
| 52 | M | 54 | 8 | 8 | T3N1M0 | IIB | G3-4 | PDAC |
| 53 | F | 74 | 12 | 4 | T3N1M0 | IIB | G2 | PDAC |
| **54** | **M** | **67** | **1** | **0** | **T2N1M0** | **IIB** | **G2** | **PDAC** |
| 55 | M | 70 | 8 | 8 | T2N0M0 | IB | G1 | PDAC |
| 56 | M | 77 | 12 | 8 | T3N1M0 | IIB | G1 | PDAC |
| 57 | M | 68 | 12 | 8 | T3N0M0 | IIA | G2 | PDAC |
| 58 | M | 60 | 8 | 12 | T2N0M0 | IB | G3 | PDAC |
| 59 | M | 70 | 4 | 2 | T3N1M0 | IIB | G2 | PDAC |
| 60 | M | 62 | 0 | 0 |  |  |  | CP |
| 61 | M | 68 | 8 | 12 | T1N0M0 | IA | G2 | PDAC |
| 62 | M | 79 | 8 |  | T1N0M0 | IA | G2 | PDAC |
| 63 | M | 61 | 2 | 4 | T3N1M0 | IIB | G2 | PDAC |
| 64 | M | 69 | 12 | 4 | T3N1M1 | IV | G2 | PDAC |
| 65 | M | 51 | 12 | 8 | T3N1M0 | IIB | G2 | PDAC |
| 66 | M | 72 | 8 | 0 |  |  |  | CP |
| 67 | M | 64 | 12 | 8 | T3N1M0 | IIB | G1 | PDAC |
| 68* | M | 51 | 6 | 12 | T4NXM0 | III |  | PDAC |
| 69 | M | 53 | 3 | 12 | T3N1M0 | IIB | G2 | PDAC |
| 70 | M | 68 | 8 | 12 | T2N1M0 | IIB | G1-2 | PDAC |
| 71* | M | 61 | 12 | 12 | T4N1M0 | III | GX | PDAC |
| 72 | M | 25 | 8 | 0 |  |  |  | autoimmune pancreatitis |
| 73 | M | 65 | 0 | 0 |  |  |  | CP |
| 74 | M | 70 | 8 | 8 | T3N1M0 | IIB | G2-3 | PDAC |
| 75 | M | 77 | 12 | 8 | T3N0M0 | IIA | G1 | PDAC |
| 76 | M | 61 | 12 | 12 | T2N1M0 | IIB | G2 | PDAC |
| 77 | M | 66 | 8 | 1 | T1N0M0 | IA | G2 | PDAC |

PDAC: Pancreatic ductal adenocarcinoma. CP: Chronic pancreatitis. **Bold** (41, 44, 54): PDAC cases with 0 points (false negatives). ***Bold cursive*** (15, 31): CP cases with high score (false positives). * Cases with no Whipple/pancreatectomy procedure (36, 40, 68 and 71): Diagnosis through biopsy of pancreatic mass.

**Table S4. Discrimination between PDAC (pancreatic ductal adenocarcinoma) and CP (chronic pancreatitis) using pathologist score.**

| **Parameters** | **pAb proCOL11A1 77 cases (54 *vs*. 23) Cut-off > 0** | **mAb proCOL11A1 69 cases (51 *vs*. 18) Cut-off > 1** |
| --- | --- | --- |
| Sensitivity | 98 % (90.1 - 100.0) | 92% (81.1 - 97.8) |
| Specificity | 83 % (61.2 - 95.0) | 83% (58.6 - 96.4) |
| False Positives | 17% | 17% |
| False Negatives | 2 % | 8% |
| + Predictive Value | 93 % | 94 % |
| - Predictive Value | 95 % | 79 % |
| Accuracy | 94 % | 90 % |

**Table S5. Summary statistics of immunohistochemical analyses**

| **Parameter** | **Mean** | **SD** | **Median** | **Range** | **P** |
| --- | --- | --- | --- | --- | --- |
| Nº + cells *PDAC* | 310.21 | 219.46 | 251.50 | 42-832 | <0.001 |
| *CP* | 61.44 | 46.64 | 53.50 | 4 - 147 |  |
| StSurface *PDAC* | 4,633.02 | 4,217.05 | 3,276.75 | 301.16 -20,648.04 | <0.001 |
| *CP* | 465.42 | 519.63 | 266.29 | 4.688 - 1,756.67 |  |
| RefArea *PDAC* | 31,188.46 | 46,570.50 | 8,939.00 | 1,444 - 128,540 | <0.001 |
| *CP* | 112,554.56 | 43,680.71 | 128,540 | 315 - 128,540 |  |
| Nº + cells/mm^2^ *PDAC* | 50,669.26 | 47,685.25 | 40,751.34 | 334.54 - 180,690 | 0.056 |
| *CP* | 19,209.98 | 52,035.75 | 443.46 | 31.12 - 174,840 |  |
| Nº + cells/RefArea *PDAC* | 0.0507 | 0.0477 | 0.0408 | 0.0003 - 0.181 | 0.056 |
| *CP* | 0.0192 | 0.0520 | 0.0004 | 0.000 - 0.175 |  |
| StSurface/RefArea *PDAC* | 0.632 | 0.4507 | 1.0000 | 0.0045 - 1.0000 | 0.001 |
| *CP* | 0.128 | 0.3404 | 0.0020 | 0.0000 - 1.0000 |  |
| Saturation *PDAC* | 110.91 | 11.50 | 112.03 | 87.50 - 128.01 | 0.970 |
| *CP* | 110.77 | 11.21 | 109.36 | 92.95 - 125.45 |  |

PDAC (pancreatic ductal adenocarcinoma), 24 cases; CP (chronic pancreatitis), 16 cases. The saturation index, an indicator of the quality of imaging processing, is similar in both diseases. ANOVA test was applied for significances.

**Table S6. Area under the ROC curve (AUC) of image analysis parameters (pancreatic ductal adenocarcinoma) PDAC vs. CP (chronic pancreatitis)**

| **Parameter** | **AUC** | **Standard error** | **P (Area=0.5)** |
| --- | --- | --- | --- |
| Area | 0.802 | 0.0753 | 0.0001 |
| **Nº + cells** | **0.901** | 0.0486 | 0.0001 |
| **Staining surface** | **0.964** | 0.029 | 0.0001 |
| Nº + cells/Area | 0.852 | 0.0598 | 0.0001 |
| Staining surface/Area | 0.893 | 0.0506 | 0.0001 |
| Nº + cells/mm^2^ | 0.857 | 0.0588 | 0.0001 |

The best AUCs are highlighted in **bold**

**Table S7. Discrimination between PDAC (pancreatic ductal adenocarcinoma) and CP (chronic pancreatitis) using various diagnostic markers in tissues.**

| **Immunostaining** | **Sample size PDAC/CP** | AUC | **Sensitivity%** | **Specificity%** |
| --- | --- | --- | --- | --- |
| Mesothelin^1^ | 18/17 |  | 100 | 94 |
| MUC1^2^ | 24/11 |  | 96 | 94 |
| MUC4^3^ | 74/19 |  | 77 | 78 |
| p53^3^ | 74/19 |  | 60 | 88 |
| Smad4^3^ | 74/19 |  | 63 | 88 |
| Maspin^3^ | 74/19 |  | 90 | 67 |
| RCAS1^4^ | 20/5 |  | 100 | 60 |
| c-erbB-2^5^ | 36/13 |  | 42 | 92 |
| EGFR^5^ | 36/13 |  | 50 | 92 |
| TGF-β1^5^ | 36/13 |  | 44 | 92 |
| KOC^6^ | 26/38 |  | 96 | 89 |
| G6PDH activity^7^ | 28/11 |  | 100 | 100 |
| COL11A1(current study) | 51/18 | 0.936 | 92 | 83 |

References

1. Hassan R *et al*. Mesothelin is overexpressed in pancreaticobiliary adenocarcinomas but not in normal pancreas and chronic pancreatitis. *Am J Clin Pathol* 2005; 124: 838-845.

2. Chhieng DC *et al.* MUC1 and MUC2 expression in pancreatic ductal carcinoma obtained by fine-needle aspiration. *Cancer (Cancer Cytopathol)* 2003; 99: 365-371.

3. Bhardwaj A *et al*. Double immunohistochemical staining with MUC4/p53 is useful in the distinction of pancreatic adenocarcinoma from chronic pancreatitis: a tissue microarray-based study. *Arch* *Pathol Lab Med* 2007; 131: 556-562.

4. Akashi T *et al*. Expression and diagnostic evaluation of the human tumor-associated antigen RCAS1 inpancreatic cancer. *Pancreas* 2003; 26: 49-55.

5. Zhang L, Yuan S-Z. Expression of c-erbB-2 oncogene protein, epidermal growth factor receptor, and TGF-β1 in human pancreatic ductal adenocarcinoma. *HBPD Int* 2002;1: 620-623.

6. Yantiss RK *et al*. KOC (K homology domain containing protein overexpressed in cancer): a novel molecular marker that distinguishes between benign and malignant lesions of the pancreas. *Am J Surg Pathol 2005*; 29: 188-195.

7. Van Driel BE *et al.* Differential diagnosis of chronic pancreatitis and pancreatic cancer in brush cytology specimens. *Cancer Res* 1999; 59: 5732-5736.

**Table S8. Discrimination PDAC (pancreatic ductal adenocarcinoma) vs. CP (chronic pancreatitis) with anti-proCOL11A1 mAb by components of score and by total score.**

|  | **Field score (F) 0-4 points** | **Staining Intensity(SI) 0-3 points** | **Total score (F x SI) 0-12 points** |
| --- | --- | --- | --- |
| CP, 18 cases (mean±SD) | 0.611 ± 1.335 | 0.222 ± 0.428 | 0.611 ± 1.335 |
| PDAC, 52 cases (mean±SD) | 3.389 ± 1.464 | 1.942 ± 0.938 | 7.327 ± 4.038 |
| P (PDAC vs. CP) | <0.0001 | <0.0001 | <0.0001 |
| AUC | 0.895 | 0.933 | 0.936 |
| Criterion (discriminant cut-off) | >1 | >0 | >1 |
