# Supplementary material for: Overexpression of COL11A1 by Cancer-Associated Fibroblasts: Clinical Relevance of a Stromal Marker in Pancreatic Cancer
Source: PLoS One. 2013 Oct 23;8(10):e78327. doi: 10.1371/journal.pone.0078327 (PMC3808536; doi:10.1371/journal.pone.0078327)
Supplement: File S2 — Global Gene Expression Analysis. Generation of a rabbit polyclonal antiserum to the variable region of human procollagen 11A1 (anti-proCOL11A1 pAb). SDS-PAGE and Western-blots. (DOCX) [file pone.0078327.s002.docx]

SUPPORTING INFORMATION

### Global Gene Expression Analysis

## Materials and Methods

We used Affymetrix GeneChips for global gene expression analysis. Freshly removed tissue samples were immediately snap-frozen in liquid nitrogen in the operating room and stored at –80 ºC until processing. Total RNA from normal pancreas (n=2), normal pancreatic ducts (n=2), chronic pancreatitis (n=2) and pancreatic ductal adenocarcinoma (PDAC) samples (n=10) was prepared using TRIzol reagent (Life Technologies, Carlsbad, USA) and purified using RNeasy columns (Qiagen, Hilden, Germany). Biotinylated cRNA was synthesized from each RNA sample and individually hybridized to the Human Genome U133A chip. Chips were stained, washed, and scanned following standard protocols. Signal intensities were normalized and filtered to select a set of genes for expression profiling. Genes specifically overexpressed in PDAC samples were identified using GCOS 1.2 (Affymetrix, Santa Clara, USA) and GeneSpring GX (Silicon Genetics, Redwood City, CA) software.

**Results**

The expression of 21,744 transcripts (20,537 well characterized genes and 1,207 expressed sequence tags) was analysed. We found 116 genes overexpressed in tumor samples compared to normal pancreas and chronic pancreatitis (Table S1). Among those, COL11A1 gene showed one of the highest and most significant overexpression in tumor samples, and was selected for further study.

**Generation of a rabbit polyclonal antiserum to the variable region of human procollagen 11A1 (anti-proCOL11A1 pAb)**

**Antigens and antibodies**

Recombinant human proCOL5A1 and pneumococcal PLY; anti-human proCOL5A1 6A7 and 18G5 mAbs and anti-PLY PLY-7 mAb; and anti-PLY pAb rabbit IgG, were obtained as already described12,14-16. Recombinant human COL11A1-T protein, anti-human COL11A1 pAb and mAb were obtained as follows.

**Sequence analysis and identification of an immunogenic region highly specific for proCOL11A1**

Collagen sequences were compared using the Multialin v5.4.1 program17. Sequence comparison among procollagens (types 1 to XVII) shows a high homology among procollagens α1 of types V and XI and α2 of type XI. To develop a polyclonal antiserum of the maximum specificity to human proCOL11A1, a region of the lowest homology between the two most similar isoforms, proCOL5A1 and proCOL11A1, was sought. Using the Laling v2.0 program18, the comparison algorithm identified the 363-393 amino acid stretch of proCOL11A1 as the most divergent with proCOL5A1 (Fig. S1, supporting information). On the other hand, a hydrophobicity analysis of the pro-COL11A1 molecule, combining ProtScale (http://www.expasy.ch/tools/ protscale.html) and the algorithm of Kyte and Doolittle, identified the 350-400 amino acid stretch as a hydrophilic region19. Finally, the sequence from amino acid residue E(268) to E(400) was chosen to generate a hydrophilic and highly specific immunogen of human proCOL11A1. Thus, this 133 amino acid long stretch is within the VAR subdomain of the N-terminal propeptide of proCOL11A1.

**Construction, expression and purification of recombinant COL11A1-T-**

**GST fusion**

In vitro COL11A1-T gene synthesis. DNA sequence codifying a highly specific and hydrophilic fragment of COL11A1 protein (COL11A1-T) was synthesised by LCR-PCR method20. The set of overlapping oligonucleotides for LCR reaction was designed using Gene2Oligo software21. LCR reaction was followed by a second PCR using modified primers which generated new EcoRI and PstI restriction sites at both ends of the PCR product.

Cloning. Synthetic COL11A1-T DNA was digested using EcoRI and PstI restriction enzymes and ligated into pET41a (Novagen) expression vector. JM109 (Promega) competent cells were transformed with pET41a/COL11A1-T construct and plated on LB+Kan agarose plates. Plasmid DNA from positive colonies was sequence verified and used for COL11A1-T protein expression.

Fusion protein expression. BL21-AI (Invitrogen) competent cells were transformed and grown in LB+Kan medium. Fusion protein expression was induced using 1mM IPTG and 0.2% L-Arabinose. Cells were harvested and protein extracts were obtained using BugBuster Protein Extraction Kit (Novagen). Identity of the purified recombinant protein was verified by western-blotting using anti-GST tag mAb (Novagen),

Recombinant COL11A1-T protein purification. The recombinant fusion protein (COL11A1-T-GST) was purified by affinity chromatography using GST-Bind Resin Chromatography Kit (Novagen) and dialyzed against phosphate-buffered saline. Purity of the recombinant protein and yield of the purification method was monitored by analysing protein extracts at every step by SDS-PAGE and Bradford assay.

**Anti-human COL11A1-T rabbit polyclonal serum production and IgG**

**purification**

For immunization, the purified COL11A1-T-GST fusion protein was emulsified 1:1 in incomplete Freund´s adjuvant. A New Zealand white rabbit was intramuscularly injected at 2-week intervals with 2 ml of immunogen emulsion. Two weeks after the last immunization, the animal was anesthetized and subjected to exanguination by cardiac puncture.

The resulting antiserum was extensively depleted of the anti-GST reactivity by chromatography on GST-agarose columns. The IgG fraction was purified on Protein A-Sepharose (anti-COL11A1- T pAb rabbit IgG).

**SDS-PAGE and Western-blots**

All experiments were performed according to standard protocols. To study potential cross-recognition of human proCOL5A1 and COL11A1-T, antigen samples were subjected to 6% polyacrylamide SDS-PAGE under reducing conditions and, subsequently, electrotransferred onto TransBlot Transfer Medium nitrocellulose membranes (BIO-RAD). The membranes were then either incubated with anti-PLY or anti-COL11A1-T pAb rabbit IgG (1µg/sample lane) and then with alkaline phosphatase-conjugated goat anti-rabbit IgG (Sigma); or with mouse monoclonal antibody PLY-7, 6A7 or 18G5 (5 μg /sample lane) and then with alkaline phosphatase-conjugated goat anti-mouse γ chain (Sigma). Finally, the Western blots were developed using the 1-StepTM NBT/BCIP substrate, ready for use (Pierce). To establish the best experimental conditions, we used recombinant PLY, PLY-7 IgG1, kappa mAb and anti-PLY pAb rabbit IgG as negative controls.
